# Supplementary material for: APOE genotypes differentially remodel the astrocytic lipid droplet-associated proteome to shape lipid droplet dynamics
Source: bioRxiv. 2025 Aug 20:2025.08.19.669163. Preprint. [Version 1] doi: 10.1101/2025.08.19.669163 (PMC12393358; doi:10.1101/2025.08.19.669163)
Supplement: 1 [file NIHPP2025.08.19.669163v1-supplement-1.pdf]

bioRxiv preprint doi: https://doi.org/10.1101/2025.08.19.669163; this version posted August 20, 2025. The copyright holder for this preprint (which was not certified by peer review) is the author/funder, who has granted bioRxiv a license to display the preprint in perpetuity. It is made available under aCC-BY 4.0 International license.

Supplementary Figures

Figure S1 - Cuní-López et. al

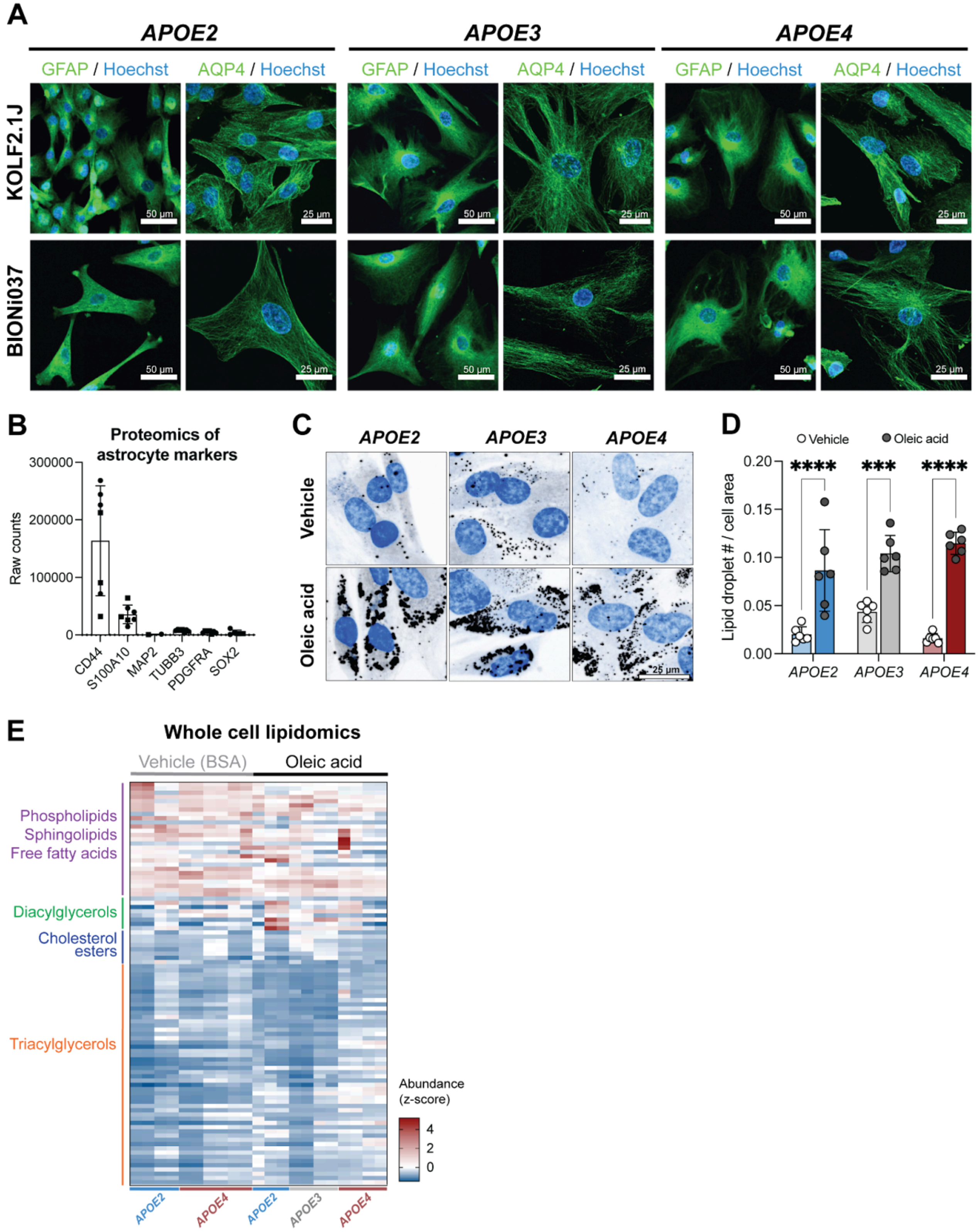

bioRxiv preprint doi: https://doi.org/10.1101/2025.08.19.669163; this version posted August 20, 2025. The copyright holder for this preprint (which was not certified by peer review) is the author/funder, who has granted bioRxiv a license to display the preprint in perpetuity. It is made available under aCC-BY 4.0 International license.

Figure S1. Data relating to Figure 1

A. iPSC-derived astrocytes from KOLF2.1J (top) and BIONi037 (bottom) isogenic series both stain for the canonical astrocytic markers, GFAP and AQP4. Scale bars are 25 µm for AQP4 and 50 µm for GFAP.
B. Raw abundance counts of protein markers for astrocytes (CD44 and S100A10) and non-astrocytic marker proteins (neurons–MAP2, TUBB3; oligodendrocytes–PDGFRA; and iPSCs–SOX2). Measurements were derived from whole cell proteomics data. Square symbols represent KOLF2.1J-derived lines and circles represent BIONi037-derived lines. Each point represents data from an independent line and derivation, n=3-4.
C. Immunofluorescence of neutral lipids (LipidSpot, black) in isogenic iPSC-derived astrocytes (BIONi037-derived) homozygous for three common *APOE* genotypes in the presence of either vehicle (BSA) or 80 µM oleic acid. Blue staining is Hoechst 33258 (nuclei). Scale bar is 25 µm.
D. Quantification of lipid droplets per cell area in isogenic iPSC-derived astrocytes (BIONi037-derived) homozygous for three common *APOE* genotypes in the presence of either vehicle (BSA) or 80 µM oleic acid. Data represent n=6 independent treatments, represented as mean ± SD. *** $P \leq 0.001$,**** $P \leq 0.0001$ by two-way ANOVA with post-hoc Šídák’s test.
E. A heatmap depicting mass spectrometry lipidomics measurements (z-score of abundance normalized to total lipid content) on unfractionated “Whole Cell” samples when cells are treated with vehicle (BSA) or oleic acid (80 µM). N=10-11 independent cell growths across two isogenic sets of human iPSC-derived astrocytes are shown with representation from multiple lipid classes.

bioRxiv preprint doi: https://doi.org/10.1101/2025.08.19.669163; this version posted August 20, 2025. The copyright holder for this preprint (which was not certified by peer review) is the author/funder, who has granted bioRxiv a license to display the preprint in perpetuity. It is made available under aCC-BY 4.0 International license.

Figure S2 - Cuní-López et. al

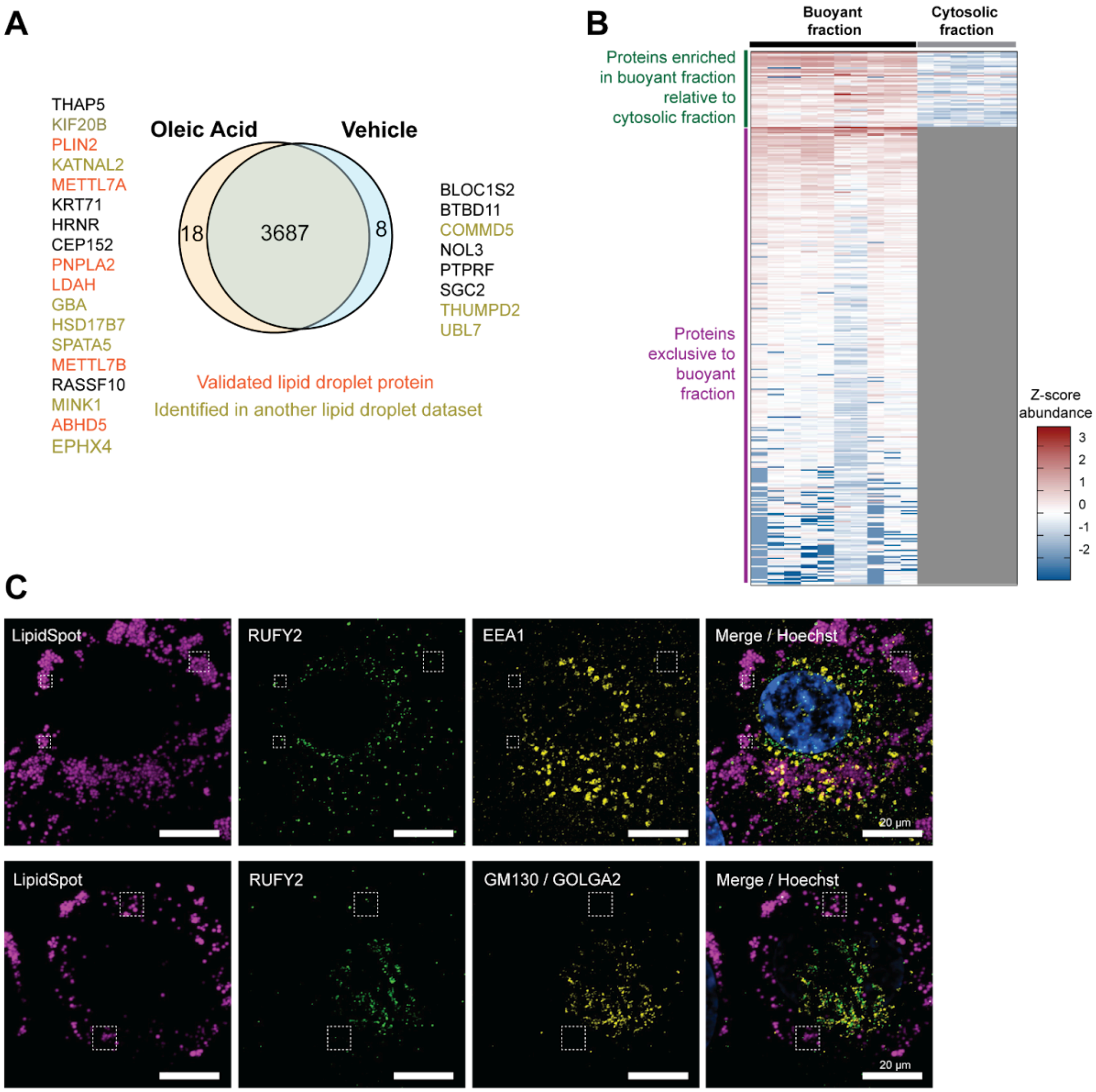

Figure S2. Data relating to Figure 2

A. Venn diagram comparing proteins present in the buoyant fractions of BSA- (vehicle) and oleic acid-treated human iPSC-derived astrocytes. Only 26 proteins were significantly differentially represented in these two treatment conditions. Orange proteins are canonical lipid droplet proteins validated by immunofluorescence in other studies. Yellow-green represents proteins identified in other published lipid droplet proteome studies from other cell types.

bioRxiv preprint doi: https://doi.org/10.1101/2025.08.19.669163; this version posted August 20, 2025. The copyright holder for this preprint (which was not certified by peer review) is the author/funder, who has granted bioRxiv a license to display the preprint in perpetuity. It is made available under aCC-BY 4.0 International license.

B. A heatmap depicting the mass spectrometry proteomics data (z-score of abundance) of proteins selected as part of our human iPSC-derived astrocyte lipid droplet-associated proteome in the buoyant fraction samples (n=10) and the cytosolic fractions (n=6).
C. Immunofluorescence staining of RUFY2 (green), lipid droplets (magenta), early endosomes (EEA1, yellow), golgi (GM130/GOLGA2; yellow) and nuclei (blue). Dashed squares show areas where RUFY2 is localized to lipid droplets but not to early endosomes or golgi. Scale bars are 20 μm.

bioRxiv preprint doi: https://doi.org/10.1101/2025.08.19.669163; this version posted August 20, 2025. The copyright holder for this preprint (which was not certified by peer review) is the author/funder, who has granted bioRxiv a license to display the preprint in perpetuity. It is made available under aCC-BY 4.0 International license.

Figure S3 - Cuní-López et. al

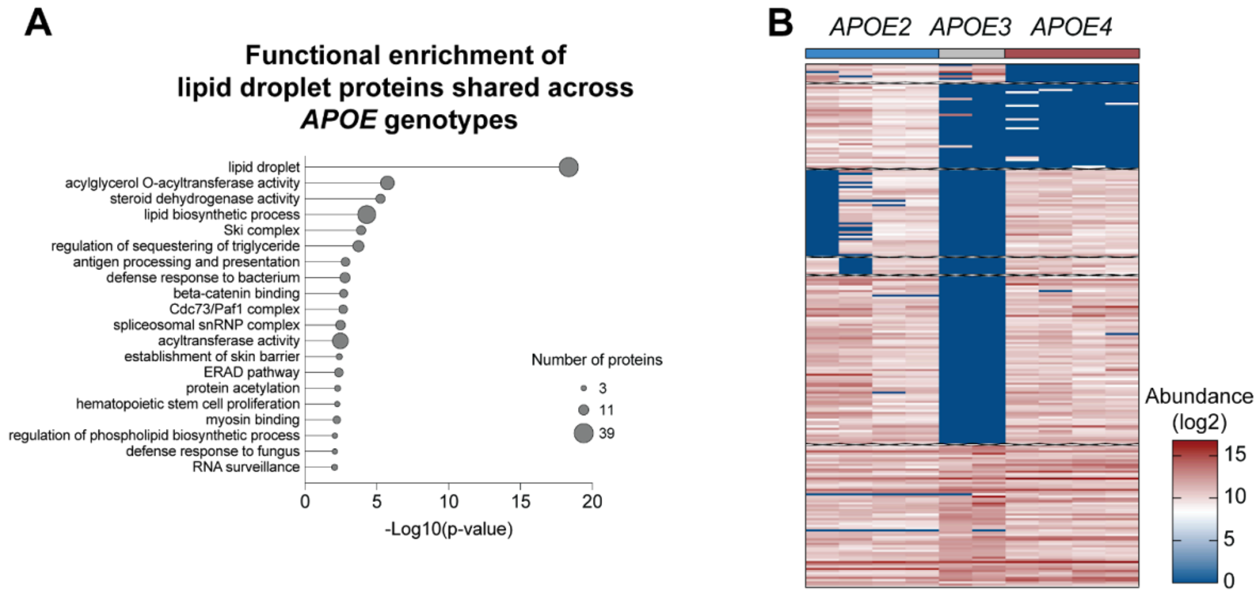

Figure S3. Data relating to Figure 3

A. Overrepresentation analysis to determine functional enrichment of lipid droplet-associated proteins commonly present in the lipid droplet-associated proteomes of all three *APOE* genotypes.
B. A heatmap depicting the mass spectrometry proteomics data (z-score of abundance) of proteins significantly differentially abundant in lipid droplets from iPSC-derived astrocytes harboring different *APOE* genotypes.

bioRxiv preprint doi: https://doi.org/10.1101/2025.08.19.669163; this version posted August 20, 2025. The copyright holder for this preprint (which was not certified by peer review) is the author/funder, who has granted bioRxiv a license to display the preprint in perpetuity. It is made available under aCC-BY 4.0 International license.

Figure S4 - Cuní-López et. al

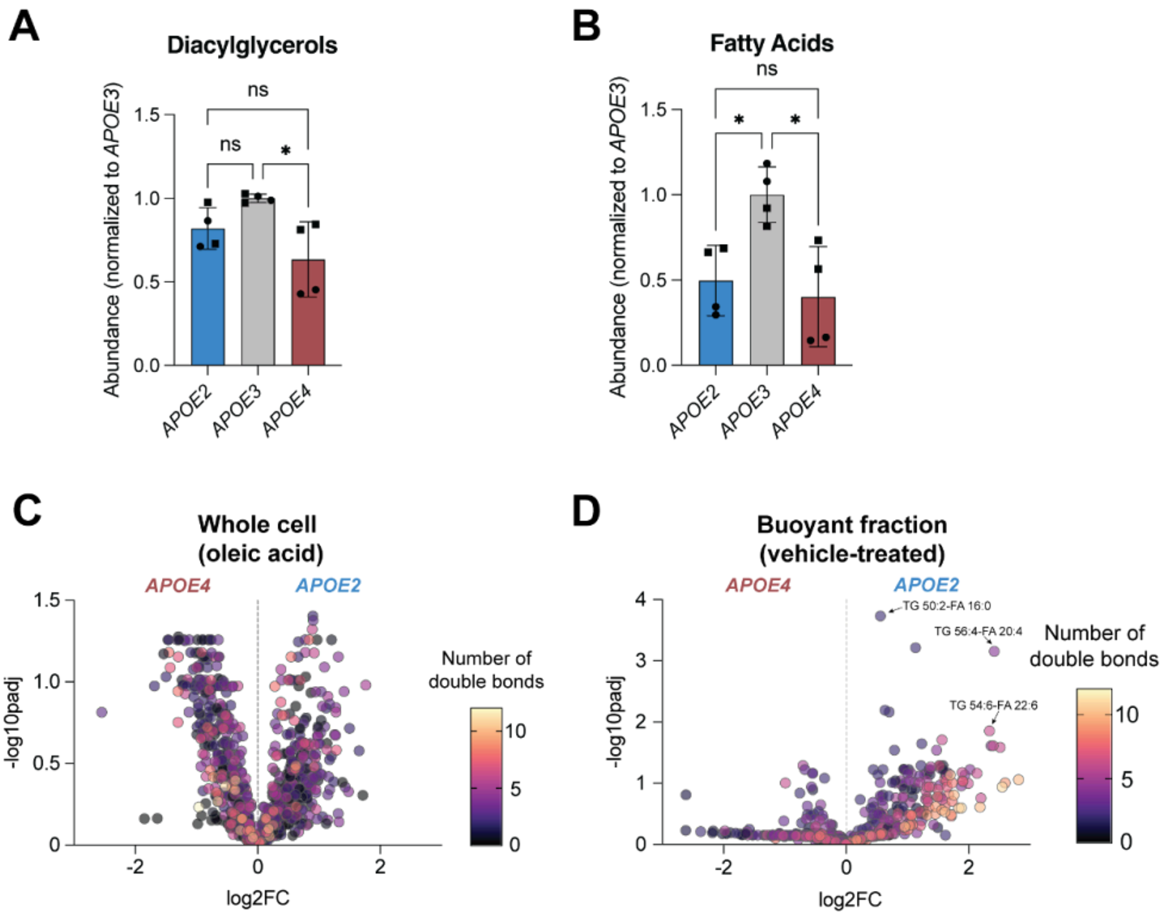

Figure S4. Data relating to Figure 4

A. Abundance of diacylglycerols in buoyant fractions of various *APOE* genotypes obtained from iPSC-derived astrocytes treated with oleic acid. Data represent n=4 independent replicates across two isogenic sets of human iPSC-derived astrocytes. * $P \leq 0.05$ by one-way ANOVA with post-hoc Tukey's test.
B. Abundance of free fatty acids in buoyant fractions of various *APOE* genotypes obtained from iPSC-derived astrocytes treated with oleic acid. Data represent n=4 independent replicates across two isogenic sets of human iPSC-derived astrocytes. * $P \leq 0.05$ by one-way ANOVA with post-hoc Tukey's test.
C. Volcano plots comparing whole cell lipids between *APOE4* and *APOE2* iPSC-derived astrocytes following treatment with oleic acid. Each data point represents individual lipid species from n=4 independent replicates across two isogenic sets of human iPSC-derived astrocytes. Color coding represents fatty acid saturation (number of double bonds).

bioRxiv preprint doi: https://doi.org/10.1101/2025.08.19.669163; this version posted August 20, 2025. The copyright holder for this preprint (which was not certified by peer review) is the author/funder, who has granted bioRxiv a license to display the preprint in perpetuity. It is made available under aCC-BY 4.0 International license.

D. Volcano plots comparing buoyant fraction lipids between *APOE4* and *APOE2* iPSC-derived astrocytes following treatment with vehicle (BSA). Each data point represents individual lipid species from n=4 independent replicates across two isogenic sets of human iPSC-derived astrocytes. Color coding represents fatty acid saturation (number of double bonds).

bioRxiv preprint doi: https://doi.org/10.1101/2025.08.19.669163; this version posted August 20, 2025. The copyright holder for this preprint (which was not certified by peer review) is the author/funder, who has granted bioRxiv a license to display the preprint in perpetuity. It is made available under aCC-BY 4.0 International license.

Figure S5 - Cuní-López et. al

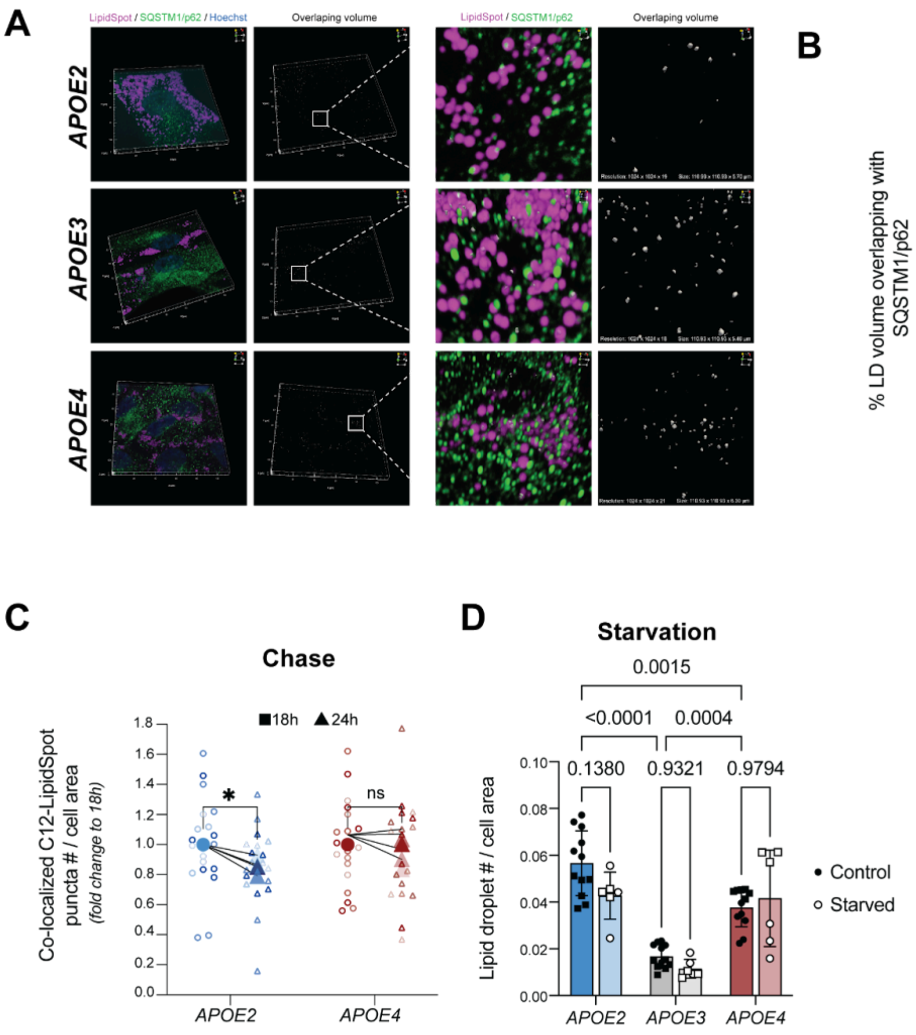

Figure S5. Data relating to Figure 5

A. Immunofluorescence staining of SQSTM1/p62 in green that contacts lipid droplets (magenta) in human iPSC-derived astrocytes harboring the three *APOE* genotypes (shown in BIONi037 line). Overlap volume is shown in white. Scale of 3D projection shown is 110.93 x 110.93 x 10-6.30 μm^3.
B. Quantification of percent of lipid droplet volume overlapping with SQSTM1/p62 signal in 3D confocal imaging (shown in BIONi037 line). Data represent quantification of an average of ~700 lipid droplets per imaging frame. Data plotted are n=3-4 imaging frames per *APOE* genotype. Data are represented as mean ± SD. **** $P \leq 0.0001$, ** $P \leq 0.01$, * $P \leq 0.05$ by one-way ANOVA with post-hoc Tukey's test.

bioRxiv preprint doi: https://doi.org/10.1101/2025.08.19.669163; this version posted August 20, 2025. The copyright holder for this preprint (which was not certified by peer review) is the author/funder, who has granted bioRxiv a license to display the preprint in perpetuity. It is made available under aCC-BY 4.0 International license.

C. The change in number of BODIPY-C_{12}-positive lipid droplets (co-labeled with LipidSpot) during a chase period of 18 h and 24 h in human iPSC-derived astrocytes harboring *APOE2* and *APOE4* genotypes (shown in BIONi037 line). Data shown in large symbols represent n=4 independent pulse chase experiments calculated as fold change to 18 h. Data shown in small symbols represent individual replicates for each experiment. * $P \leq 0.05$ by repeated measures two-way ANOVA with post-hoc Šidák's test.
D. Quantification of lipid droplets per cell area in two isogenic sets of human iPSC-derived astrocytes under regular culture conditions (control) and starvation for 5 days (starved). Each point represents an independent treatment. Data are represented as mean ± SD. **** $P \leq 0.0001$ by two-way ANOVA with post-hoc Tukey's test.
